# Global-Mode Analysis of Full-Disk Data from the *Michelson Doppler Imager* and the *Helioseismic and Magnetic Imager*

**DOI:** 10.1007/s11207-017-1201-5

**Published:** 2018-01-31

**Authors:** Timothy P. Larson, Jesper Schou

**Affiliations:** 1 Stanford University, Stanford, CA USA; 2 Max-Planck-Institut für Sonnensystemforschung, Göttingen, Germany

**Keywords:** Helioseismology, Observations, Oscillations, Solar

## Abstract

Building upon our previous work, in which we analyzed smoothed and subsampled velocity data from the *Michelson Doppler Imager* (MDI), we extend our analysis to unsmoothed, full-resolution MDI data. We also present results from the *Helioseismic and Magnetic Imager* (HMI), in both full resolution and processed to be a proxy for the low-resolution MDI data. We find that the systematic errors that we saw previously, namely peaks in both the high-latitude rotation rate and the normalized residuals of odd $a$-coefficients, are almost entirely absent in the two full-resolution analyses. Furthermore, we find that both systematic errors seem to depend almost entirely on how the input images are apodized, rather than on resolution or smoothing. Using the full-resolution HMI data, we confirm our previous findings regarding the effect of using asymmetric profiles on mode parameters, and also find that they occasionally result in more stable fits. We also confirm our previous findings regarding discrepancies between 360-day and 72-day analyses. We further investigate a six-month period previously seen in $f$-mode frequency shifts using the low-resolution datasets, this time accounting for solar-cycle dependence using magnetic-field data. Both HMI and MDI saw prominent six-month signals in the frequency shifts, but we were surprised to discover that the strongest signal at that frequency occurred in the mode coverage for the low-resolution proxy. Finally, a comparison of mode parameters from HMI and MDI shows that the frequencies and $a$-coefficients agree closely, encouraging the concatenation of the two datasets.

**Electronic Supplementary Material:**

The online version of this article (10.1007/s11207-017-1201-5) contains supplementary material, which is available to authorized users.

## Introduction

Designed to be the successor to the *Michelson Doppler Imager* (MDI: Scherrer *et al.*, 1995) onboard the *Solar and Heliospheric Observatory* (SOHO), the *Helioseismic and Magnetic Imager* (HMI: Schou *et al.*, 2012) was launched onboard the *Solar Dynamics Observatory* (SDO) in February 2010. The designs of the two instruments are quite similar; here we note the differences between the two projects that are most pertinent to global-mode analysis. HMI is equipped with a $4096 \times 4096$ pixel CCD and takes images with a spatial resolution of approximately 0.5 arscec per pixel, or about four times that of MDI. SDO is in geosynchronous orbit, whereas SOHO orbits the Sun–Earth L_1_ Lagrange point; partly for this reason, HMI is able to send down much more telemetry. Among other observables, HMI produces full-resolution dopplergrams at a cadence of 45 seconds. Last, HMI observes the Fe i 6173 Å spectral line, so that it sees a slightly lower height in the solar atmosphere than MDI, which observed the Ni i 6768 Å line (Fleck, Couvidat, and Straus, 2011).

Global-mode analysis of data from the MDI Medium-$\ell $ Program and systematic errors therein were described by Larson and Schou (2015), hereafter referred to as LS15. Before an attempt is made to extend this medium-$\ell $ analysis to HMI data, it is fitting to apply it to the MDI full-disk data and compare the results. Although one might expect the two MDI analyses to be in near-perfect agreement, our investigation reveals surprising differences. In particular, systematic errors such as the “bump” seen in the normalized residuals of the odd $a$-coefficients and the anomalous peak in the near-surface rotation rate at high latitudes have different characteristics in the analysis of full-disk data.

MDI full-disk data are available throughout the mission, but usually with a low duty cycle. Nominally, for two months per year, telemetry was allocated to send down the full-disk images continuously. These time intervals constitute the Dynamics Program. As discussed in the next section, the actual lengths of the full-disk observing campaigns varied widely across the mission, as did their timing within the year.

One might say that the primary difference between the MDI full-disk data and low-resolution data (labeled vw_V, see LS15) is that the latter are smoothed and subsampled (see Section 3), leaving them with a resolution one-fifth that of the full-disk data. However, another important difference lies in the data cropping. Whereas the vw_V data are cropped to 90% of the average solar radius onboard the spacecraft, the full-disk data extend significantly closer to the limb. Further details are provided in Section 3.

In order to provide continuity with the MDI Medium-$\ell $ Program, we used the HMI data to create a vw_V proxy. This also allowed us to further investigate periodicities seen in the $f$-mode frequencies from the analysis of MDI vw_V data.

In the next section we describe the datasets used in our analysis. In Section 3 we discuss how these data were analyzed, with emphasis on how each analysis differs from the analysis in LS15. Section 4 gives the results, first for MDI and then for HMI, followed by a comparison of the two instruments. Section 5 describes a six-month periodicity in data from both MDI and HMI and discusses the effect of $B_{0}$ (the heliographic latitude of the sub-observer point) on leakage matrices and the resulting inversions for solar rotation. Finally, in Section 6 we discuss our findings and propose how we might proceed.

## Data

Beginning in 1996, MDI was continuously operated in full-disk mode for a few months each year through 2010. We therefore have 15 time intervals to analyze, known as the Dynamics Runs. To choose the exact intervals to use for global-mode analysis, one must balance the lengths of the time series and their duty cycles. For the most part, we have followed previous investigators, notably Rabello-Soares, Korzennik, and Schou (2008) and Rhodes *et al.* (2011). In our case, the simplest criterion was maximizing mode coverage. Another factor that we considered was choosing intervals similar to each other in length in order to facilitate comparing them.

For 2000, only 45 days of continuous data were available, and for 2003 only 38 days were available. There were, however, small additional sections of continuous data for these years, separated from the previously used time intervals by sections with a low duty cycle. We therefore extended both time series. In 2002, the situation was reversed; more data were available on the other side of a large gap, but including it did not result in substantially increased mode coverage. Therefore we chose a length that was closer to the other Dynamics Runs.

The first part of Table 1 shows the time series that we used for the analysis presented here. The second part of the table shows time series used in various other investigations. In both cases, processing was carried out through the mode fitting. The time series and resulting mode parameters can be downloaded from the Stanford Joint Science Operation Center (see the Appendix for details). The exception is the 12-day long interval in 2003, which was too short for the mode fitting to succeed, so only time series are available.

**Table 1:** Dynamics time series. Day numbers refer to the first day of the time series and are given relative to the MDI epoch of 1 January 1993 00:00:00 TAI. All time series begin on the first minute of the start date and end on the last minute of the end date. Duty cycles are given for the raw time series (DC1) and the time series after gapfilling (DC2). The number of modes fitted with 6 $a$-coefficients (NM6) and with 36 $a$-coefficients (NM36) is also given. The first part of the table shows the time series used for this article; the second part shows time series used for various other investigations.

Day	Length [Days]	Start Date	End Date	DC1	DC2	NM6	NM36
1238	63	23 May 1996	24 Jul. 1996	0.93	0.98	2039	1729
1563	93	13 Apr. 1997	14 Jul. 1997	0.91	0.98	2106	1840
1834	92	09 Jan. 1998	10 Apr. 1998	0.90	0.97	2132	1862
2262	77	13 Mar. 1999	28 May 1999	0.92	0.97	2101	1809
2703	98	27 May 2000	01 Sep. 2000	0.74	0.89	2056	1770
2980	90	28 Feb. 2001	28 May 2001	0.91	0.97	2088	1837
3331	109	14 Feb. 2002	02 Jun. 2002	0.85	0.96	2092	1839
3904	76	10 Sep. 2003	24 Nov. 2003	0.58	0.75	1988	1603
4202	65	04 Jul. 2004	06 Sep. 2004	0.87	0.96	2062	1741
4558	67	25 Jun. 2005	30 Aug. 2005	0.92	0.98	2082	1755
4830	62	24 Mar. 2006	24 May. 2006	0.89	0.98	2073	1723
5454	58	08 Dec. 2007	03 Feb. 2008	0.87	0.98	2032	1687
5540	64	03 Mar. 2008	05 May 2008	0.85	0.96	2088	1740
5981	65	18 May 2009	21 Jul. 2009	0.75	0.84	2017	1631
6335	67	07 May 2010	12 Jul. 2010	0.85	0.93	2031	1704
2703^a^	45	27 May 2000	10 Jul. 2000	0.93	1.00	1919	1556
3296^b^	27	10 Jan. 2002	05 Feb. 2002	0.86	0.93	1864	1127
3331^b^	98	14 Feb. 2002	22 May 2002	0.86	0.97	2081	1821
3368^a^	72	23 Mar. 2002	02 Jun. 2002	0.90	0.97	2056	1717
3904^b^	12	10 Sep. 2003	21 Sep. 2003	0.81	0.98	0	0
3942^a^	38	18 Oct. 2003	24 Nov. 2003	0.81	0.94	1921	1367

^a^Rabello-Soares, Korzennik, and Schou (2008).

^b^E.J. Rhodes, Jr., private communication, 2017.

In order to make comparisons with the vw_V data, we used the same 15 time intervals for two other analyses. First, we used the regular vw_V data. Second, we used the full-disk images, but apodized them in the same way as the vw_V data. We also attempted to use the full-disk apodization on vw_V images that we reconstructed from the full-disk images, but this was only possible for 1996 and 1998, because for the other years the gaussian convolution kernel used for the smoothing reached beyond the full-disk crop radius, resulting in the loss of large amounts of data. These last two variations in the analysis required the computation of new leakage matrices. Details of the apodization are provided in the next section.

In all cases, we used a window function common^1^ to all analyses for each time interval as input to the gapfilling. The result was mainly to discard a large amount of the regular vw_V data. We did not repeat the analysis of the regular full-disk data using the common window function, but the original window function included at most 0.23% more data.

HMI began producing regular science data on 30 April 2010. Since that time, we have been performing medium-$\ell $ analysis of the data using 72-day long time series in phase with the original MDI medium-$\ell $ time series. The time intervals for which results are presented here are shown in Table 2. We have also created 360-day long time series by concatenating the gapfilled 72-day long time series.

**Table 2:** HMI time series. Day numbers refer to the first day of the time series and are given relative to the MDI epoch. Duty cycles are given for the raw time series (DC1) and the time series after gapfilling (DC2).

Day	Start Date	DC1	DC2	Day	Start Date	DC1	DC2
6328	30 Apr. 2010	0.996	1.000	7408	14 Apr. 2013	0.986	0.991
6400	11 Jul. 2010	0.982	0.995	7480	25 Jun. 2013	0.990	0.997
6472	21 Sep. 2010	0.968	0.995	7552	05 Sep. 2013	0.967	0.997
6544	02 Dec. 2010	0.989	0.995	7624	16 Nov. 2013	0.993	0.997
6616	12 Feb. 2011	0.963	0.991	7696	27 Jan. 2014	0.969	0.997
6688	25 Apr. 2011	0.997	1.000	7768	09 Apr. 2014	0.989	0.995
6760	06 Jul. 2011	0.987	0.997	7840	20 Jun. 2014	0.991	0.997
6832	16 Sep. 2011	0.966	0.991	7912	31 Aug. 2014	0.972	1.000
6904	27 Nov. 2011	0.990	0.997	7984	11 Nov. 2014	0.992	0.997
6976	07 Feb. 2012	0.966	0.997	8056	22 Jan. 2015	0.963	0.991
7048	19 Apr. 2012	0.998	1.000	8128	04 Apr. 2015	0.989	0.993
7120	30 Jun. 2012	0.990	0.997	8200	15 Jun. 2015	0.989	0.997
7192	10 Sep. 2012	0.971	0.997	8272	26 Aug. 2015	0.970	0.997
7264	21 Nov. 2012	0.993	0.997	8344	06 Nov. 2015	0.978	0.990
7336	01 Feb. 2013	0.972	0.997	8416	01 Jan. 2016	0.972	0.997

## Method

The MDI full-disk data were processed in almost exactly the same way as the vw_V data, that is, using the updated method described by LS15. The most notable exception is that for the full-disk data, it is possible to use a larger fraction of the input images; whereas the vw_V data are apodized with a cosine in fractional image radius from 0.83 to 0.87, the full-disk data are apodized in the same way from 0.90 to 0.95. It should also be noted that each analysis uses a leakage matrix appropriate to the data used. For the full-disk data, the leakage matrix was calculated as described by LS15, except that the input images were not convolved with anything. In particular, we did not account for any point-spread function, but this is expected to have little effect in the medium-$\ell $ regime.

In summary, all analyses of MDI data presented here were corrected for various geometric effects during spherical harmonic decomposition: image-scale errors, cubic distortion from the instrument optics, misalignment of the CCD, an error in the inclination of the Sun’s rotation axis, and a potential tilt of the CCD. The spherical harmonic time series were then detrended and gapfilled as described by LS15, and Fourier transforms of these were fit to extract the mode parameters. The fitting, or peakbagging, as it is called, took into account horizontal displacement at the solar surface and the distortion of eigenfunctions by the differential rotation (known as the “Woodard effect”: Woodard, 1989). For the regular full-disk analysis, the peakbagging was also repeated using asymmetric mode profiles in addition to the normally used symmetric profiles.

For the analysis of HMI data, the input images were already corrected for optical distortion. Hence, the only geometrical correction applied here was for the error in the inclination of the Sun’s rotation axis mentioned in the previous paragraph. After the spherical harmonic decomposition, the HMI data were processed in almost exactly the same way as the MDI full-disk data. In particular, the images were apodized in the same way, and therefore an identical leakage matrix was used. The peakbagging was performed using both symmetric and asymmetric mode profiles for the 72-day long time series and 360-day long time series.

In addition, we have created a proxy for the MDI vw_V data from the HMI data. This was done by binning the HMI data by a factor of four to simulate the MDI full-disk data, convolving them with a gaussian, and retaining only every fifth point in each direction, as described by LS15. The resulting images were then apodized in the same way as the MDI vw_V data, and the peakbagging likewise used the same leakage matrix. We fit these data only as 72-day long time series and only using symmetric profiles.

Whether we used the HMI images in their regular resolution or by way of the proxy, the most significant difference with the MDI processing was in the detrending. Whereas the MDI data needed to have discontinuities in the time series identified manually, for HMI, this information can be derived from keywords in the input data. Furthermore, the quality of the HMI data is more carefully tracked, so that the keywords also provide a reliable measure of what data are expected to be present.

Owing to its orbit and problems with calibration, the HMI spherical harmonic time series contain a strong daily oscillation. We therefore detrended them using different parameters than those used for the MDI data. Although in both cases the time series were detrended by subtracting Legendre polynomials of degree seven, for HMI these polynomials were fit to an interval of 1100 points (825 minutes), which was advanced by 960 points (720 minutes). In other words, the detrending intervals overlapped by 140 points (105 minutes). For additional details, we refer to LS15.

## Results

### MDI Mode Parameters

**Figure 1:**
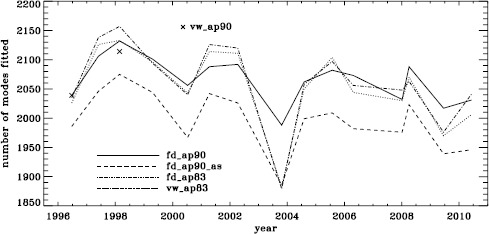
Mode coverage for all Dynamics Runs. Symbols show the number of modes fitted in 1996 and 1998 by the vw_ap90 analysis.

In total, we applied four different analyses to all 15 of the Dynamics Runs. For conciseness, we make use of the following additional labels: fd_ap90 for the full-disk analysis using its regular apodization, fd_ap90_as for the same when fit with asymmetric profiles, and fd_ap83 for the full-disk data apodized like the vw_V data. We use the label vw_ap83 when we use the vw_V with its regular apodization, but note that we processed it using a window function common to all analyses. We also use the label vw_ap90 for the vw_V data apodized like the full-disk data, but note that this analysis is only available for the 1996 and 1998 Dynamics Runs. Figure 1 shows the number of modes fitted using six $a$-coefficients (which parameterize the dependence of the frequencies on $m$, see LS15) for all of these analyses. As expected from our previous work, the mode coverage for the fd_ap90_as analysis as a function of time is basically the same as that for the fd_ap90 analysis shifted downward. Interestingly, the other two analyses are closer in coverage to the fd_ap90 analysis, with the exception of the 2003 Dynamics Run, which had the lowest duty cycle by far. Apparently, the regular full-disk analysis was less susceptible to this low duty cycle than all of the other analyses. The effect of using asymmetric profiles on the mode parameters themselves is discussed in the context of the HMI analysis.

In order to compare two different analyses, we must create common modesets. For example, in order to quantify the effect of the apodization, for each Dynamics Run we found the modes common to the fd_ap90 and fd_ap83 analyses. For each mode parameter, we then took a weighted average in time over all Dynamics Runs for which the mode was successfully fit in both analyses. For the weights, we used the length of each time series multiplied by its duty cycle. We also computed the average error, rather than the error on the average, and for comparison between two analyses, we used the larger error estimate of the two. Thus the significance that we show is the least that one might expect from an average Dynamics Run. Last, the noise parameter [$b$] required special treatment. Since $\mathrm{e}^{b}$ is proportional to the length of the time series, each background parameter had $\log (T/72.0)$ subtracted from it before averaging, where $T$ is the length of the time series in days. Except where noted, we used the fitted parameters resulting from using six $a$-coefficients.

**Figure 2:**
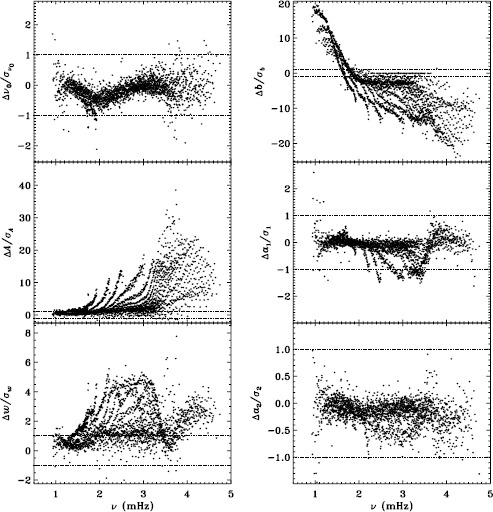
Effect of apodization on mode parameters. We show changes in frequency [$\nu _{0}$], amplitude [$A$], width [$w$], background parameter [$b$], $a_{1}$, and $a_{2}$ in units of the standard deviation. Each panel is scaled differently; *horizontal lines* show the ${\pm}\, 1 \sigma $ levels. For the $a$-coefficients, no more than nine points have been excluded from the range shown. The sense of subtraction is fd_ap90 minus fd_ap83.

In Figure 2 we show the result for six mode parameters: frequency, amplitude, width, background, $a_{1}$, and $a_{2}$. For a full explanation of these, we refer to LS15. Clearly, the most significant change is to the amplitudes. One might think that this is to be expected since the fd_ap83 data are apodized to a smaller radius, but in fact, this ought to be corrected for in the leakage matrix. In other words, the parameter $A$ should represent the intrinsic amplitude of the mode *on the Sun*. Next most significant is the change to the background, which was lower for the fd_ap83 analysis at lower frequencies, and higher at higher frequencies. The widths were lower for the fd_ap83 analysis across all frequencies, especially between 2.0 and 3.0 mHz. Last, although not very significant, the bump seen in the difference in $a_{1}$ is encouraging, since it is in the same location as the bump that we hope to eliminate.

Here we note that in the absence of systematic errors, these differences should all be small (${\ll}\,1\sigma $) near the peak power of the $p$-mode band (around 3.0 mHz), since the signal-to-noise ratio is high there (Libbrecht, 1992). In any case, the differences should have no trends in frequency or any other parameter. One source of random error, the stochastic excitation of the modes, is the same for all observers and apodizations, since the modes considered here have lifetimes that are long enough to be considered truly global. Another source of random error, convective motions on the surface, could be different when using different parts of the solar disk, but this still should not cause any offsets in the frequencies, widths, or $a$-coefficients. Although the amplitudes and background parameters could be affected, such an effect would still be flat in frequency. Even when the signal-to-noise ratio is low, the changes should still be random. Hence, we can already see that there is a problem with the analysis.

**Figure 3:**
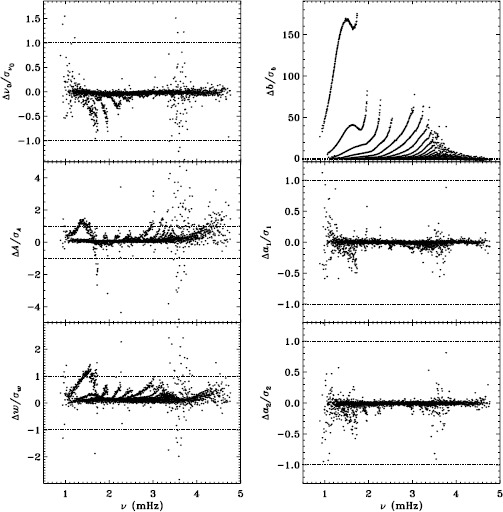
Effect of smoothing and subsampling on mode parameters. We show changes in frequency [$\nu _{0}$], amplitude [$A$], width [$w$], background parameter [$b$], $a_{1}$, and $a_{2}$ in units of the standard deviation. For clarity, the *bottom panels* have at most 0.65% of points excluded. Each panel is scaled differently; *horizontal lines* show the $\pm 1 \sigma $ levels. The sense of subtraction is fd_ap83 minus vw_ap83.

To quantify the effect of smoothing and subsampling, we compare the fd_ap83 and vw_ap83 analyses in exactly the same fashion. Figure 3 shows the results. Here the convective noise is the same, as well as any instrumental effect, since the two datasets observe almost the same part of the solar disk. Indeed, with the exception of the background parameter, the smoothing and subsampling results in smaller changes than the apodization. The average of the other parameters shows almost no significance at all. In particular, the differences in the $a$-coefficients are hardly different from zero, which would suggest that the smoothing and subsampling have little effect on any inversion results.

**Figure 4:**
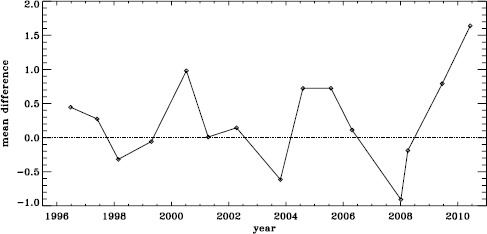
Effect of smoothing and subsampling on amplitudes. We show the mean changes in units of the standard deviation for all Dynamics Runs. The sense of subtraction is fd_ap83 minus vw_ap83.

The small difference for the amplitude shown in Figure 3 is, however, deceptive. For all other parameters, the differences look roughly the same for the different Dynamics Runs, but for the amplitudes, the difference actually alternates in sign. This is shown in Figure 4, where we have plotted the mean significance as a function of time. We have no explanation for this oscillation so far, but focus and tuning changes in the instrument are likely candidates.

### Systematic Errors in MDI data

**Figure 5:**
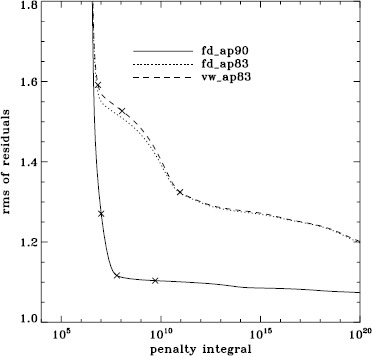
Tradeoff curves for an average over all Dynamics Runs. Symbols, from *left* to *right*, indicate tradeoff parameters of $\mu =10^{-4}$, $\mu =10^{-6}$, and $\mu =10^{-9}$.

To explore the effect of the different analyses on our systematic errors, we began by performing simple one-dimensional regularized least-squares (RLS) rotational inversions using the $a_{1}$-coefficient alone, just as in LS15. In this case, we formed mode sets common to all three of the fd_ap90, fd_ap83, and vw_ap83 analyses for each Dynamics Run, and we took the temporal average as before, except that for inversions we always used the error on the average. The tradeoff curves in Figure 5 show the result. The curve for the fd_ap90 analysis has the shape that one hopes to see: a single “elbow”, so that one may unambiguously choose a tradeoff parameter, not to mention that the $\chi^{2}$-values are closer to unity. It is satisfying to see that the value typically used, $\mu =10^{-6}$, lies right where it should on the curve: “the place where the residuals stop decreasing sharply, so that further decreases of $\mu $ will be of little benefit” (LS15). The other two curves are very close to the final curve that we found in LS15, and we have marked the tradeoff parameters that we used previously.

**Figure 6:**
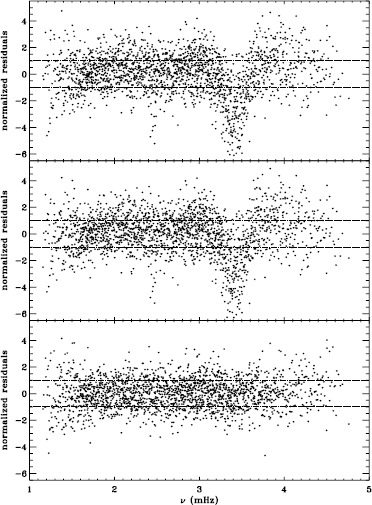
Normalized residuals of $a_{1}$ for an average over all Dynamics Runs. We show from *top* to *bottom* the vw_ap83 analysis, the fd_ap83 analysis, and the fd_ap90 analysis. *Horizontal lines* show the ${\pm}\, 1 \sigma $ levels.

**Figure 7:**
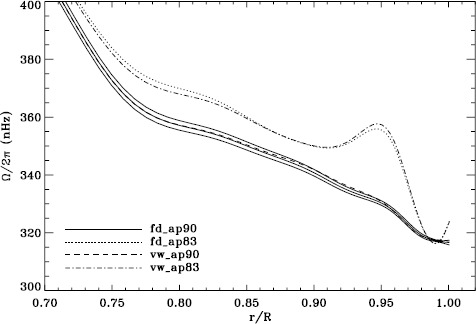
Internal rotation as a function of radius at $75^{\circ }$ latitude for four analyses applied to the 1998 Dynamics Run. *Solid lines* show the fd_ap90 analysis and its error; errors for the other analyses were similar.

In order to see how the different analyses affect our inference of how the solar rotation varies with latitude, we performed two-dimensional RLS inversions using 36 $a$-coefficients. First, we formed averages over the Dynamics Runs just as we did for the one-dimensional inversions. The residuals of $a_{1}$ resulting from inversions of these averages are shown in Figure 6. As one can see, the analyses using the vw_V apodization clearly show the bump, whereas it is essentially absent from the fd_ap90 analysis. Investigating the polar jet (LS15), we found that it was clearly visible in inversions of the 1998 Dynamics Run alone, so we are able to compare all four analyses. Again, we took the mode set common to all four. As Figure 7 shows, we again see that using a smaller apodization radius results in the polar jet, while the larger apodization radius shows no sign of it. Here we must reiterate that the bump does not cause the jet; previous research has shown that excluding from the inversions the modes that constitute the bump still shows the jet (Schou *et al.*, 2002). Hence, for both the bump and the jet, we are left with a puzzle. Using the vw_V apodization results in both of the systematic errors, which are then removed by using *more* data from the input images, although the data added are expected to contain only a small fraction of the helioseismic signal. The most likely explanation is an error in the analysis codes or leakage matrix, but so far, no error explaining our results has been found.

### HMI Mode Parameters

**Figure 8:**
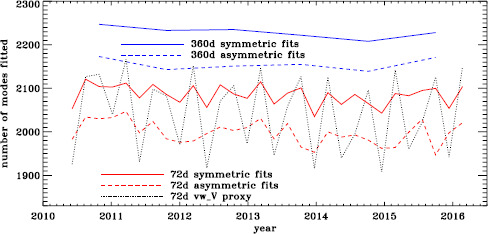
Number of modes fitted as a function of time for the first six years of HMI measurements.

So far, we have analyzed about six years of HMI data as 72-day and 360-day fits for the full-disk data, using both symmetric and asymmetric profiles. For the vw_V proxy, we used only 72-day long time series and symmetric profiles. The resulting number of modes fitted is shown in Figure 8. The difference in coverage between symmetric and asymmetric fits and between 360-day fits and 72-day fits is what we have come to expect based on our analysis of other datasets. The large oscillation in coverage of the fits to the vw_V proxy data is surprising, however, especially since it exceeds the coverage of the full-disk fits at its peak. We return to this fact below.

**Figure 9:**
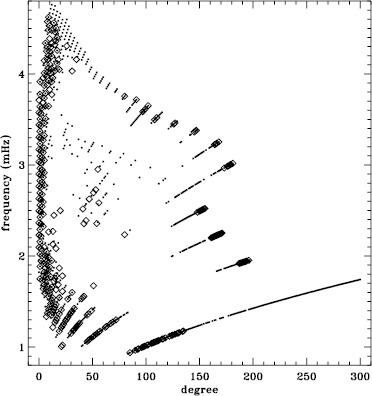
Difference in mode coverage for the first six years of HMI measurements. Diamonds show modes that failed to fit at least once with symmetric profiles when asymmetric profiles succeeded, and dots show the opposite.

**Figure 10:**
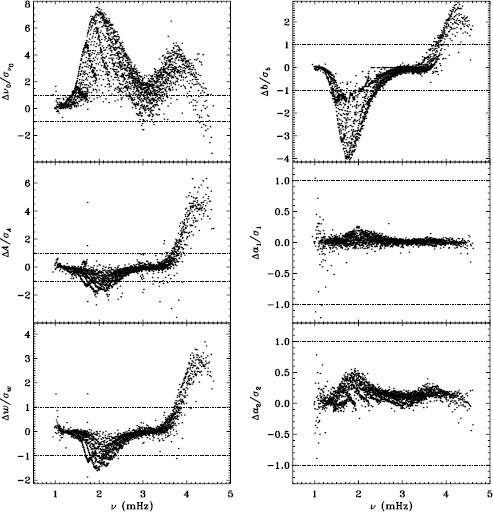
Effect of asymmetric profiles on mode parameters from 72-day fits. We show changes in frequency [$\nu _{0}$], amplitude [$A$], width [$w$], background [$b$], $a_{1}$, and $a_{2}$ in units of the standard deviation. The data have been averaged over six years of HMI measurements. Each panel is scaled differently; *horizontal lines* show the $\pm 1 \sigma $ levels. At most, 0.18% of points have been excluded. The sense of subtraction is asymmetric minus symmetric.

In LS15, we found that the fits using asymmetric profiles are much less stable than those using symmetric profiles. This is not surprising, since the asymmetric fits require an extra parameter, but it does result in decreased mode coverage. However, in the region where the modes are observed to have strong asymmetry, one must accept that using asymmetric profiles more accurately characterizes them. Hence, the parameters resulting from both types of fitting have become standard data products. The difference in coverage for the 72-day fits is shown in Figure 9, where diamonds indicate a mode that failed at least once using symmetric profiles when asymmetric profiles succeeded, and dots indicate a mode that failed at least once using asymmetric profiles when symmetric profiles succeeded. The difference in mode parameters themselves are shown in Figure 10, where we have performed averaging in the same manner as before, using the 72-day fits. This figure is to be compared to the last panel of Figures 4 – 8 in LS15. Clearly, fitting asymmetric profiles has a large effect on the resulting frequencies in a range between 1.0 and 3.0 mHz. The other mode parameters were similarly, but less significantly, affected in a slightly smaller frequency range, still centered at about 2.0 mHz. For the amplitudes, widths, and background parameters, there was also a large and opposite change above 3.8 mHz, while the frequency differences show a second peak around the same frequency. Although not shown here, we found similar differences using the MDI full-disk data. Hence, we can be sure that the asymmetry of the modes is characterized in the same way by all of the datasets that we studied.

Unfortunately, this also means that the error magnification that we saw for the frequencies and background parameters in LS15 is also present in the analysis of the full-disk datasets.

**Figure 11:**
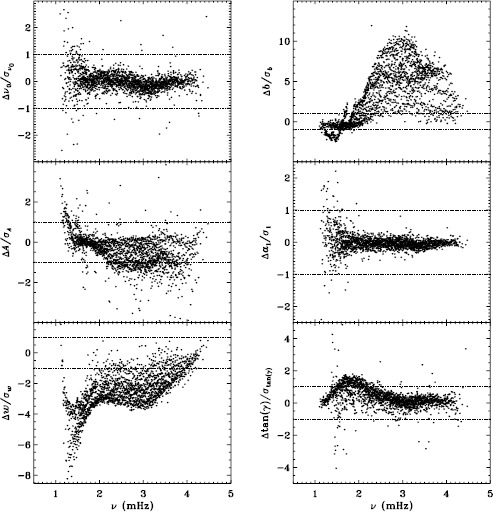
Difference between 360-day and 72-day fits in frequency [$\nu _{0}$], amplitude [$A$], width [$w$], background [$b$], $a_{1}$, and tangent of the asymmetry parameter [$\gamma $] in units of the standard deviation. The data have been averaged over six years of HMI measurements. Each panel is scaled differently; *horizontal lines* show the $\pm 1 \sigma $ levels. At most, 0.66% of points have been excluded. The sense of subtraction is 360-day long fits minus 72-day long fits.

Our previous work also revealed discrepancies between 360-day fits and an average over 72-day fits for the MDI vw_V data, regardless of whether symmetric or asymmetric profiles were used. To confirm that this reflects a characteristic of the algorithm and not the data, we repeated the comparison for the first six years of HMI. Figure 11 shows the results using asymmetric profiles. Comparison with Figure 13 of LS15 reveals the same trends. The exception is the amplitude differences, but this can be attributed to the gaussian smoothing applied to the vw_V data. Although not shown here, we also found error ratios similar to those shown in LS15. This would indicate that the difference has to do with the algorithm and not with the data. However, Barekat, Schou, and Gizon (2014) found significant differences between the two instruments in the radial gradient of the rotation rate at high latitudes near the surface. In subsequent investigations, Barekat (private communication, 2015) also found that the results using the 360-day fits for the HMI differed significantly from those using the averaged 72-day fits, while for MDI the two are essentially in agreement. Clearly, further study is needed to determine the source of these differences.

### Systematic Errors in HMI data

**Figure 12:**
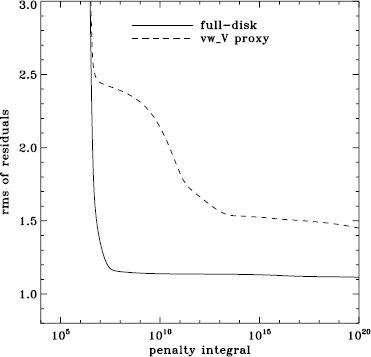
Tradeoff curves for an average over six years of HMI measurements.

**Figure 13:**
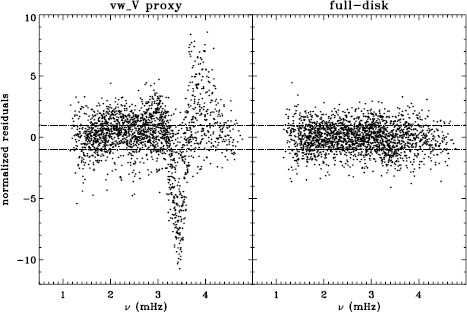
Normalized residuals of $a_{1}$ for an average over six years of HMI measurements. The *left panel* shows the HMI vw_V proxy. The *right panel* shows the full-disk HMI analysis. *Horizontal lines* show the ${\pm}\, 1 \sigma $ levels.

**Figure 14:**
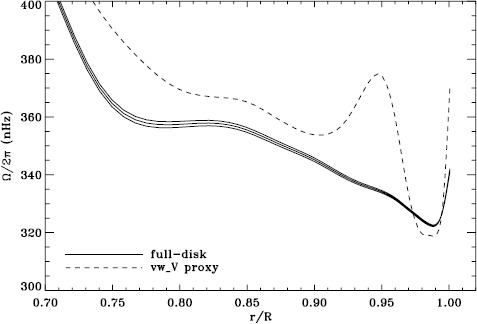
Internal rotation as a function of radius at $75^{\circ }$ latitude for an average over six years of HMI measurements. The *solid lines* show the full-disk analysis and its error; errors for the other analysis were similar.

We plot tradeoff curves and normalized residuals of $a_{1}$ for the HMI full-disk and vw_V proxy analyses, shown in Figures 12 and 13, in the same way as for the MDI data. Comparison reveals similar differences between the full-disk and low-resolution results as for MDI. The tradeoff curve shows higher residuals, and the bump in the residuals of $a_{1}$ is much more significant. For the rotation rate at high latitudes, the HMI temporal coverage allowed us to discover that the jet is only discernible when $|B_{0}|$ is maximum, although the two analyses still resulted in significantly different rotation rates. Furthermore, the upturn in the rotation rate near the surface at $75^{\circ }$ is more pronounced at these times for the vw_V proxy. When $B_{0}$ is close to zero, we see the upturn in both analyses, but it is stronger for the vw_V proxy. Both features are clearly seen in an average over the six years that we have analyzed, shown in Figure 14.

### Comparison of MDI and HMI

**Figure 15:**
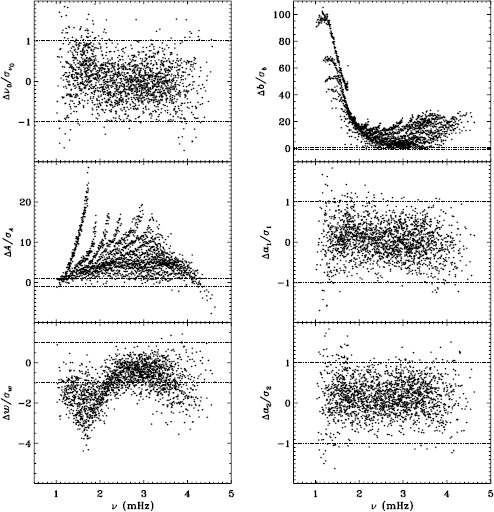
Difference between HMI and MDI full-disk fits for the 2010 Dynamics Run. Each panel is scaled differently; *horizontal lines* show the ${\pm}\, 1 \sigma $ levels. The sense of subtraction is HMI minus MDI.

**Figure 16:**
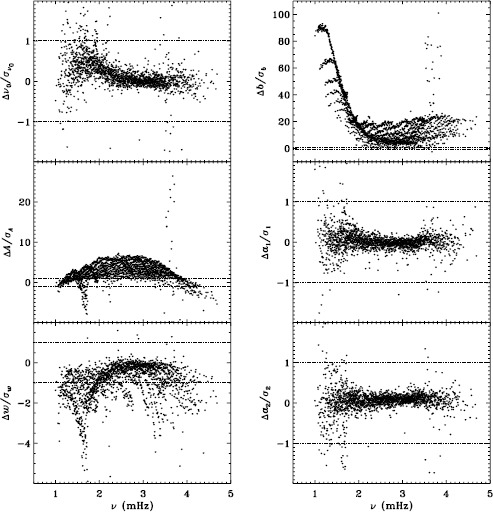
Difference between fits to the HMI vw_V proxy and MDI vw_V data for the first 72 days of HMI measurements. Panels are scaled as in Figure 15 to facilitate comparison, with *horizontal lines* showing the ${\pm}\, 1 \sigma $ levels. The sense of subtraction is HMI minus MDI.

MDI and HMI were both operating during the 2010 Dynamics Run. Hence, we have the opportunity to compare the mode parameters resulting from each dataset. Unfortunately, since the two instruments operate at two different cadences, it is not straightforward to generate a common window function. Setting this aside, Figure 15 shows a comparison of the modes common to the fd_ap90 and regular HMI analyses for this time interval using their original window functions. Similarly, Figure 16 compares the analysis of the HMI vw_V proxy and the MDI vw_V datasets for the first 72 days of HMI. Again, since realization noise is identical for the two instruments, we hope to see small differences for the frequencies, widths, and $a$-coefficients, since these parameters should not depend on the formation height of the respective absorption lines used for the observations.

These figures are encouraging in that the frequencies and $a$-coefficients do show little change between the two instruments, although there is a hint of a feature in the frequency differences around 1.7 mHz. One is not surprised to see large differences in amplitude and background parameter, since these parameters do depend on the height at which the mode is observed. The fact that the amplitude differences are not the same in Figures 15 and 16 may be explained by the different center-to-limb dependence of the observing height for the two instruments. Unfortunately, the widths observed by the two instruments are not consistent, with HMI systematically measuring lower values.

**Figure 17:**
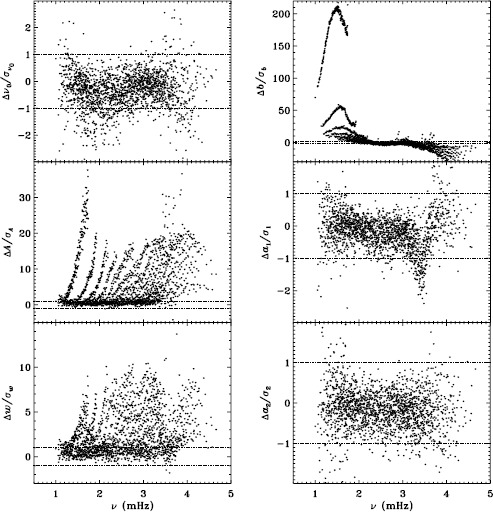
Difference between HMI full disk and vw_V proxy analyses for the first 72 days of HMI measurements. Each panel is scaled differently; *horizontal lines* show the ${\pm}\, 1 \sigma $ levels. The sense of subtraction is full-disk minus vw_V proxy.

**Figure 18:**
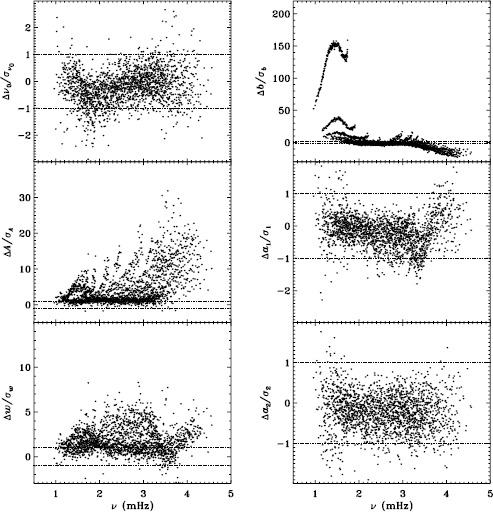
Difference between the MDI fd_ap90 and vw_ap83 analyses for the 2010 Dynamics Run. Panels are scaled as in Figure 17 to facilitate comparison, with *horizontal lines* showing the ${\pm}\, 1 \sigma $ levels. The sense of subtraction is fd_ap90 minus vw_ap83.

To see how much of the discrepancy results from differences in the instruments and how much results from differences in the processing, Figure 17 plots the difference between the HMI full-disk fits and the fits to the vw_V proxy data for the first 72 days of HMI measurements, while Figure 18 plots the difference between the fd_ap90 and vw_ap83 for the 2010 Dynamics Run. In other words, Figure 18 can be thought of as the sum of Figures 2 and 3 for a single Dynamics Run. The close similarity of Figures 17 and 18 gives us confidence that the observed differences have little to do with the source of the data.

## Effect of $B_{0}$

### Six-Month Periodicity

**Figure 19:**
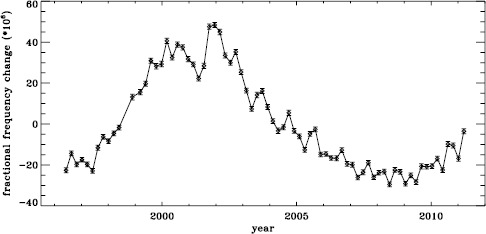
Fractional change in $f$-mode frequency for the entire MDI mission.

**Figure 20:**
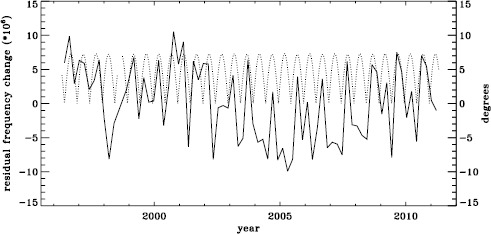
Fractional change in $f$-mode frequency for MDI with the solar-cycle dependence removed. The absolute value of $B_{0}$ is overplotted (*dotted line*).

The original analysis of the vw_V data revealed a one-year period in the fractional frequency change of the $f$-modes. In LS15, we found that the amplitude of the annual component increased with increasing degree, but it was decreased by correcting for the Doppler shift that is caused by the motion of SOHO relative to the Sun. In Figure 19 we show the fractional change in $f$-mode frequency for the entire MDI mission using the most recent fitted mode parameters resulting from using symmetric profiles and 36 $a$-coefficients. The values shown were averaged over a range in $\ell $ from 251 to 300 and corrected for Doppler shift. To see how the frequency shifts vary with the solar cycle, we plotted them against the average rms value of the line-of-sight magnetic field, as given by the DATARMS keyword in the corresponding data series.^2^ We found a linear relationship between the two and subtracted it. Now, rather than a one-year period, we predominantly see a six-month period, presumably related to the absolute value of $B_{0}$. To demonstrate that this is so, we overplot the two quantities in Figure 20. The correlation coefficient between the frequency shifts and the average absolute value of $B_{0}$ is 0.42.

**Figure 21:**
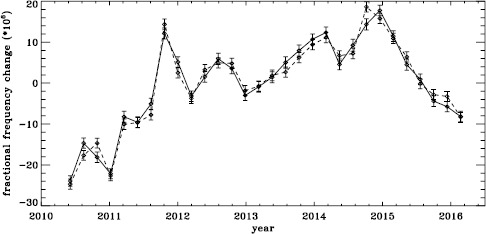
Fractional change in $f$-mode frequency for the first six years of HMI measurements. The *solid line* shows the vw_V proxy, and the *dashed line* shows full-disk data.

**Figure 22:**
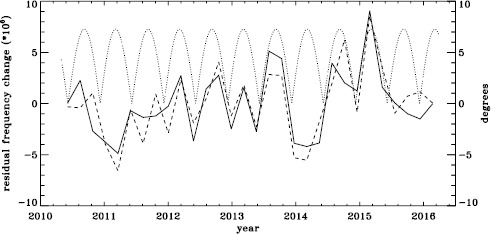
Fractional change in $f$-mode frequency for HMI with the solar-cycle dependence removed. The *solid line* shows the vw_V proxy, and the *dashed line* shows full-disk data. The absolute value of $B_{0}$ is overplotted (*dotted line*).

**Figure 23:**
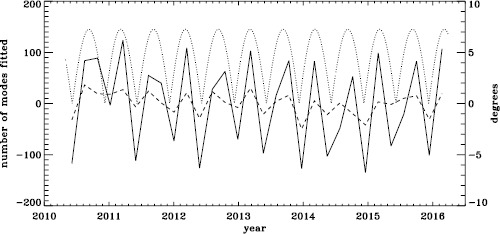
Number of modes fitted as a function of time for the HMI full-disk (*dashed line*) and the vw_V proxy (*solid line*) relative to their means (total number fitted shown in Figure 8). The absolute value of $B_{0}$ is overplotted (*dotted line*).

To see if the same is true for HMI, we first applied the same procedure to the vw_V proxy, although in this case the motion of the spacecraft relative to the Sun has already been corrected for in the dopplergrams by shifting their target times. To see how the smoothing, subsampling, and apodization might affect the frequency shifts, we repeated this for the HMI full-disk data. The result is shown in Figure 21, where we see that the two analyses almost always agree within their errors. In each case, we then subtracted a linear function of the average magnetic field, as before. For the vw_V proxy, we again see a prominent six-month signal, but it is slightly weaker than for MDI, as Figure 22 shows. In this case, the correlation coefficient was 0.39. For the full-disk data, the correlation was only 0.28. However, inspection of the number of modes fitted as a function of time for the vw_V proxy, shown in Figure 8, reveals exactly this period. Overplotting the absolute value of $B_{0}$ further reveals that contrary to all expectation, mode coverage is lowest when $|B_{0}|$ is minimum, as Figure 23 shows. Here the correlation coefficient is 0.95. For completeness, we note that the correlation when using the full-disk data is only 0.78. When we recall that the leakage matrix was computed assuming $B_{0}=0$, it can only come as a shock that we fit more modes when the leakage matrix is most incorrect. Until this discovery, one might have thought that the variation of mode parameters with $B_{0}$ was related to the approximation that the leaks from $\Delta \ell + \Delta m$ odd are zero, since it assumes north–south symmetry. It now seems much more likely that the variation has to do with which part of the solar surface is visible.

### Leaks for Maximum $|B_{0}|$

**Figure 24:**
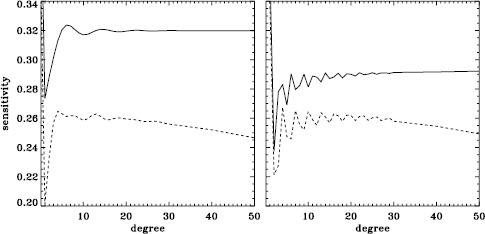
Sensitivity to target mode; the *left panel* shows $m=0$, and the *right panel* shows $m=\ell $. The *solid lines* show the original leakage matrix, and the *dashed lines* show leaks for high $|B_{0}|$ ($=6.35^{\circ }$).

**Figure 25:**
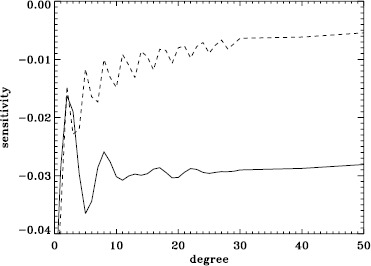
Leaks for $\Delta \ell =1$, $\Delta m =0$, and high $|B_{0}|$ ($=6.35^{\circ }$). The *solid line* shows $m=0$, and the *dashed line* shows $m=\ell $. The original leaks are identically zero.

A variation in the analysis suggested by the results of the previous section is to use a leakage matrix for a non-zero $B_{0}$. By good fortune, $B_{0}$ was near its minimum in the middle of the 1998 Dynamics Run, its average value being $-6.35^{\circ }$. We repeated the peakbagging for this interval using full-disk leakage matrices computed for this value of $B_{0}$ for both apodizations. We must point out, however, that the results using the new leakage matrices are not necessarily any more correct than the original results, since in both cases, the leaks from $\Delta \ell + \Delta m$ odd are ignored. Phrased another way, the leakage-matrix elements that we used became more accurate, but those that we ignored became different from zero. To illustrate the relative magnitude of the odd leaks, we plot sensitivities to the target mode ($\Delta \ell = \Delta m = 0 $) in the two cases in Figure 24. In Figure 25, we plot odd elements of the new leakage matrices. For the sake of brevity, we show only the real part of the radial component of the leakage matrix.

**Figure 26:**
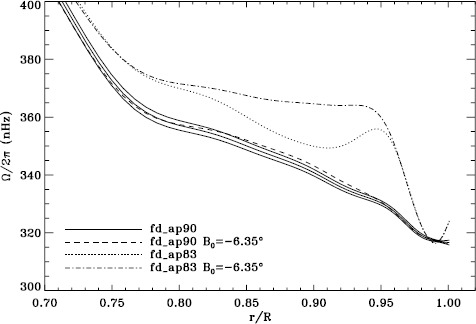
Effect of leakage matrix on inversions. We show internal rotation as a function of radius at $75^{\circ }$ latitude for four analyses applied to the 1998 Dynamics Run. Two of the curves were shown in Figure 7. The *solid lines* show the fd_ap90 analysis and its error; errors for the other analyses were similar. For these inversions, the full mode sets were used, rather than common mode sets.

**Figure 27:**
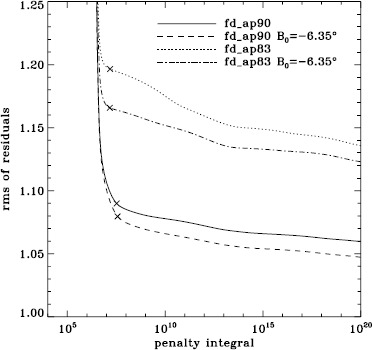
Effect of leakage matrix on residuals. We show tradeoff curves for four analyses applied to the 1998 Dynamics Run. Symbols indicate a tradeoff parameter of $\mu =10^{-6}$. For these inversions, the full mode sets were used, rather than common mode sets.

Although this is not shown, we found that the mode parameters changed similarly for the two apodizations. The unsurprising exception was that the change in $a_{1}$ showed the bump, with marginal significance, when using the vw_V apodization. The amplitudes and background parameters showed highly significant changes, while the changes in width were moderately significant. The results of two-dimensional RLS inversions are shown in Figure 26. Clearly, a large change resulted between 0.83R_⊙_ and 0.95R_⊙_ when the vw_V apodization was used, whereas the change when the full-disk apodization was used was not significant. Although this is not shown, we also found similar results using the smoothed data. Plotting the tradeoff curves, shown in Figure 27, we see that the new leakage matrix resulted in lower residuals for both of the apodizations.

## Discussion and Prospects

In comparing the MDI full-disk data with the vw_V data, we found that the difference in mode parameters, with the exception of the background, mostly resulted from the different apodizations used in the two analyses. In particular, the difference in $a_{1}$ showed the bump at 3.4 mHz. Correspondingly, two-dimensional RLS inversions of data using the full-disk apodization did not show the bump in the residuals, whereas it appeared almost the same in the two analyses using the vw_V apodization. Likewise, the high-latitude jet was almost completely absent when using the full-disk apodization. In one-dimensional inversions, the tradeoff curve for the full-disk analysis using the vw_V apodization still showed the anomalous shape seen in LS15.

**Figure 28:**
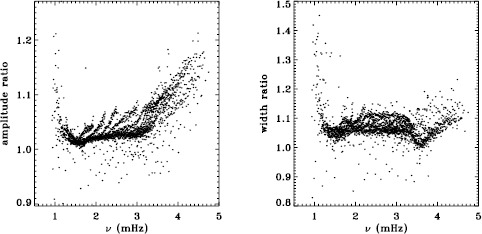
Ratios of amplitude and width from the fd_ap90 analysis to those from the fd_ap83 analysis for an average over all Dynamics Runs. For the width, 17 points have been excluded from the range shown.

To further explore the possible cause of these discrepancies, we plotted the ratio of the amplitudes from the full-disk analysis using its regular apodization to the amplitudes found using the vw_V apodization, and likewise for the widths. The result is shown in Figure 28. The shape of these ratios is roughly the same as the differences shown in the second and third panels of Figure 2, which were plotted in units of significance. The difference in amplitudes would suggest a problem with the leakage matrix, which could also affect the widths, but these differences might also be attributed to the model that we used for the background. Although not shown, we found that the background differences themselves also showed a trend similar to that seen in the significance.

Smoothing and subsampling made highly significant changes only to the background parameter. Recalling that $\mathrm{e}^{b}$ multiplies the covariance of the noise at high frequencies (LS15), one might guess that the gaussian convolution somehow changes the noise in that range. The smoothing and subsampling also made significant changes to the amplitude, and these changes varied in sign across the Dynamics Runs. One probable cause for the sign change is the difference between the best focus and the commanded focus in the instrument, which varied throughout the mission. The occasional changes in the instrument tuning to compensate for drifts of the optical elements are also likely to play a part. The question of how the smoothing and subsampling change the amplitude at all remains unanswered, as their effect should be accounted for in the leakage matrix. In the future, one might perform the smoothing without subsampling, since subsampled data should result in greater interpolation errors when the images are remapped, which could account for some of the differences. Other methods of smoothing and subsampling are possible, as well as measuring the covariance of the noise in different frequency intervals.

The analysis of HMI data confirmed that using a proxy for the vw_V data resulted in both the high-latitude jet and the bump in the odd $a$-coefficients, whereas both were essentially absent from the analysis of full-disk data. Comparison of fits using asymmetric mode profiles to those using symmetric profiles revealed differences similar to those seen in LS15 and in the analysis of MDI full-disk data. In spite of fitting fewer modes, asymmetric profiles (occasionally) resulted in more stable fits at the ends of ridges, mostly at the low-$\ell $ ends, but also at the high-$\ell $ ends for $p$-modes of low to moderate radial order. Comparison of 360-day fits to an average of 72-day fits also revealed differences similar to those seen in LS15. Other investigators (Barekat, Schou, and Gizon, 2016), however, have found differences in the inversions of mode sets from the two instruments, which we have not discussed here, but which should be investigated in the future.

HMI also allows us to compare the difference between the full-disk results and those for the vw_V proxy in the magnitude of the six-month oscillation. Although we did not examine the frequency shifts for the full-disk data, we found the surprising result that more modes were fitted for the vw_V proxy when the absolute value of $B_{0}$ was at its peak. This might suggest that the systematic errors that we see are related to the alignment of the apodization circles with the spherical harmonic node lines. To see if this is true, one might try using differently shaped apodizations, such as apodizing in longitude and latitude rather than image radius, or an elliptical apodization.

**Figure 29:**
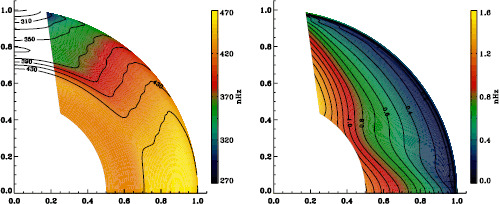
Internal rotation (*left*) and the corresponding errors (*right*) derived from the MDI full-disk analysis averaged over all Dynamics Runs. We have erased color from the regions where estimates of rotation are deemed unreliable; contours are retained on the left for ease of labeling.

**Figure 30:**
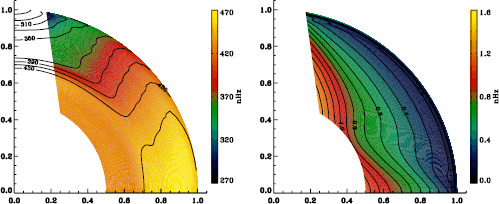
Internal rotation (*left*) and the corresponding errors (*right*) derived from an average over the first six years of the HMI 72-day analysis. We have erased color from the regions where estimates of rotation are deemed unreliable; contours are retained on the left for ease of labeling.

In the comparison of mode parameters from HMI and MDI, we found that differences in frequencies and $a$-coefficients were not significant for the full-disk analyses, and even less so for the vw_V analyses. While the frequency differences indicated a small feature, the differences in $a$-coefficients were almost completely flat. Since these are the only parameters used in rotational inversions, there should be no problem with concatenating datasets from the two instruments in order to increase the interval over which consistent physical inferences can be drawn. As an example, Figures 29 and 30 show internal rotation derived from full-disk datasets for MDI and HMI, respectively. Following Schou *et al.* (1998), we have removed the region where estimates of rotation are deemed unreliable. As expected, the two inferences agree quite well.

Furthermore, assuming that the full-disk analyses are more accurate than the vw_V analyses, we can use the former to correct the latter. This is essential for MDI, since the vw_V data are the only helioseismic dataset it provided with a high duty cycle.

### Electronic Supplementary Material

Below are the links to the electronic supplementary material.

(TXT 1 kB)

(TAR 362.4 MB)

(TAR 230.0 MB)

## Appendix

Detailed information on how to access MDI data from the global helioseismology pipeline can be found on the website of the Joint Science Operations Center (JSOC) at jsoc.stanford.edu/MDI/MDI_Global.html and likewise for HMI at jsoc.stanford.edu/HMI/Global_products.html. These pages contain documentation describing how the datasets used in this article were made and how they can be remade. A description of these data and their keywords was also given in the Appendix of LS15; data formats and keyword names remain unchanged in this work. All mode parameters presented here, as well as the rotational inversions shown in Figures 29 and 30, are available in the Electronic Supplementary Material. The data series from which the relevant data may be downloaded are described below.

Mode parameters resulting from both symmetric and asymmetric fits to regular full-disk data from MDI can be found in two data series: mdi.fd_V_sht_modes and mdi.fd_V_sht_modes_asym. Mode parameters for the nonstandard analyses can be found in su_tplarson.mdi_V_sht_modes. In all cases, the first primekey [T_START] should be specified as an MDI day number, found in Table 1, suffixed by “d”. Since some of the time series have the same start times, in general one must also specify NDT, the number of points in the time series (see Table 1; an MDI time series has 1440 points per day). For the nonstandard analyses, the TAG keyword should also be specified; it can take values of fdvwap, vwcomm, and vwfdap corresponding to the labels fd_ap83, vw_ap83, and vw_ap90 used in this article.

For HMI, all mode parameters presented here reside in the official HMI name space hmi. The data series are hmi.V_sht_modes and hmi.V_sht_modes_asym for the full-disk data, and the day numbers are found in Table 2. For the former data series, there is also a record corresponding to the last Dynamics Run. Since these series also contain both 72-day and 360-day fits, the primekey NDT should also be specified; note that an HMI time series has 1920 points per day. For the vw_V proxy, the data series is hmi.vw_V_sht_modes.

Newly available online are data series containing the results of two-dimensional RLS inversions for rotation. These series have the same primekeys as those containing the mode parameters and three more in addition: NACOEFF, RADEXP, and LATEXP. NACOEFF is the number of $a$-coefficients used in fitting the mode parameters, RADEXP is the exponent of the radial tradeoff parameter ($=10^{\mathsf{RADEXP}}$), and LATEXP is likewise the exponent of the latitudinal tradeoff parameter. To date, only values of $\mathsf{RADEXP}=-6$ and $\mathsf{LATEXP}=-2$ have been used, and these are also the default values for these keywords. NACOEFF can take values of 6, 18, or the default of 36. The data available include the rotation profile, its errors, and the output $a$-coefficients. The data series names are the same as those given for mode parameters above, with the string modes replaced by 2drls. For a full explanation of the format of these data, we refer to the Electronic Supplementary Material or the above websites. Note that at this time, all online inversions have used full modesets.

For the mode parameters and inversions in the MDI and HMI namespaces used in this article, the VERSION keyword is set to version2. Furthermore, all of the intermediate data products are also available (archived), and the data series names can be found on the above websites. For the nonstandard analyses, the gapfilled time series and window functions are archived; the data series are su_tplarson.mdi_XXX_V_sht_gf and su_tplarson.mdi_XXX_V_sht_gf_gaps, where XXX can be one of fdvwap, vwcomm, or vwfdap as above. The raw time series and window functions have not been archived, but can be recreated if needed.

## Footnotes

1 The window function is a time series of ones and zeros designating good and bad data points, respectively. The common window function is the product of two or more other corresponding window functions.

2 For MDI, this data series is mdi.fd_M_96m_lev182, which, as the name implies, samples the magnetic field at a cadence of 96 minutes. We looked at all available records for each 72-day interval and rejected outliers above 100 Gauss. The average was taken over the remaining records. For HMI, the data series is hmi.M_720s, which has a cadence of 12 minutes. We therefore took every eighth record to give a sampling similar to that of MDI, and no outlier rejection was needed.
