# Supplementary material for: Global-Mode Analysis of Full-Disk Data from the Michelson Doppler Imager and the Helioseismic and Magnetic Imager
Source: Sol Phys. 2018 Jan 31;293(2):29. doi: 10.1007/s11207-017-1201-5 (PMC6566312; doi:10.1007/s11207-017-1201-5)
Supplement: Supplementary file 2 — (TAR 362.4 MB) [file 11207_2017_1201_MOESM2_ESM.tar › HMI/Global_products.html]

 


Joint Science Operations Center (JSOC) Data Products


Your browser does not support JavaScript, or has "active content" blocked; Menu system will not function.

Joint Science Operations Center (JSOC)
 


---

Match ALL words
Match ANY word

  
HMI Data Products

- HMI Observables
  - LOS Magnetograms
  - LOS Dopplergrams
  - Continuum Intensity
  - Linewidth
  - Linedepth
  - HMI Products Overview
- Magnetic Field Products
  - Magnetic Master Page
  - LOS Magnetograms
  - Vector Magnetic Field Data
  - Synoptic Maps
  - HARPS
- Helioseismology Products
  - Rings
  - Time Distance Products
  - Global Helioseismology Products
  - Farside Helioseismic Monitor
- Lower Level Data Products
  - Filtergrams

AIA Data Products

- AIA Data Products
- Level 1 Data- AIA Lev1 Overview

MDI Data Products

- MDI Observables
  - MDI Filtergrams
  - MDI Dopplergrams
  - MDI Continuum Intensity
  - MDI LOS Magnetograms
  - MDI Linedepth
- Magnetic Field Products
  - MDI LOS Magnetograms
- MDI Helioseismology Products
  - MDI Local Helioseismology Products
  - MDI Global Helioseismology Products
- MDI Calibration Data
  - MDI Calibration

SHA Data Products

- GONG Data
- TON Data

IRIS Data Products

- IRIS

SID Data Products

- SID

- Data Access
  - Getting Data
  - FAQ
  - Data products to Data series Map
  - Exportdata
  - Register Email
  - Lookdata
  - Script Driven
  - VSO
- Visual Catalog
  - Latest HMI Images
  - Latest AIA Images (Sun Today)
  - Browse HMI Images/Movies
  - Browse AIA JPEG2000 Images
  - Browse AIA Fits Images
  - HMI Image Gallery
  - AIA Image Gallery
  - MDI Image Gallery
- Docs
  - Wiki
  - AIA SDO Docs
  - AIA Data Analysis Guides
  - JSOC Overview
  - JSOC Software Doc
  - Software Repository
  - Problem Reports
- News & Events

## Global Helioseismology Products

*Global helioseismology is the study of resonant modes of
oscillation inside the Sun and infers properties of the interior.*

#### Algorithm for calculation

The global helioseismology pipeline begins with a spherical harmonic decomposition. Dopplergrams are the usual input, but other observables can be used as well. Images are remapped to a uniform grid in longitude and sin(latitude), which provides an opportunity to make various geometric corrections. The resulting map is then apodized in fractional image radius from 0.90 to 0.95, and an inner product is taken with a set of target spherical harmonic masks, yielding a complex amplitude as a function of spherical harmonic degree l and azimuthal order m for each image, up to l=1000. For each l and m a timeseries is constructed, typically of length 72 days, which are then detrended and gapfilled, at which point power spectra are also computed. Fourier transforms of the detrended and gapfilled timeseries up to l=300 are fitted to yield the medium-l mode parameters. The mode parameters can be inverted to yield the Sun's internal rotation and sound speed.

Since we cannot see the entire Sun, the spherical harmonic decomposition is not able to perfectly separate the modes. The extent to which each mode leaks into its neighbors is quantified by the leakage matrix. A separate leakage matrix is required for full disk data and the MDI vw\_V proxy, but in each case the same leakage matrix has been used for all times for the standard analysis.

For the full global helioseimology documentation (for developers), see here. For a complete scientific description of the pipeline, see this paper.

#### Keywords

Except for the last two dataseries below, all of the dataseries used in the globalhs pipeline have T\_START as their first primekey, which is typically slotted with a width of one day and an epoch of 1993.01.01\_TAI, the MDI epoch (see the DRMS dataset names documentation). When applicable, the next two primkeys will be LMIN and LMAX, although for some products in the pipeline these will be equal (they are retained for consistency among the dataseries). The last primekey will generally be NDT, the number of points in the corresponding timeseries. Dataseries may optionally contain a final primekey TAG, which is simply a string which serves to label different processing options. For keywords not described here, see the JSOC keywords document.

- T\_START (time) - the beginning of the time interval a record corresponds to, specified as a date string or as an offset from the MDI epoch 1993.01.01\_TAI.
- LMIN (integer) - minimum spherical harmonic degree represented.
- LMAX (integer) - maximum spherical harmonic degree represented.
- NDT (integer) - number of time points represented.
- T\_STEP (float) - length of a time step in seconds, equal to the CADENCE keyword of the input data. This value is usually 60.0 and always a constant. The length of the timeseries is then NDT times T\_STEP.
- T\_STOP (time) - the beginning of the following timeseries, or T\_START + NDT \* T\_STEP.
- T\_OBS (time) - the midpoint of a timeseries as given by (T\_START + T\_STOP) / 2.
- MAPMMAX (integer) - maximum azimuthal order m in the remapping, usually constant.
- SINBDIVS (integer) - number of increments in sin(latitude) in the remapping, usually constant.
- MFLIPPED (integer) - a constant describing the sign convention for azimuthal order m: 0 for the MDI convention, 1 for the GONG convention.
- TAG (string) - an arbitrary string which serves to make records unique when their other primekeys are equal.
- VERSION (string) - an arbitrary string which facilitates searching obsolete versions of records.
- QUALITY (int) - a 16 digit bitfield specifying various aspects of data quality. Top bit set means no data. Bottom bit set means means mixed values of CALVER64 in the input data.
- DATE (time) - the time at which the record was created.

Dataseries for inversions have these three primekeys as well:

- NACOEFF (integer) - number of a-coefficients used in fitting the mode paramters.
- RADEXP (integer) - exponent of the radial tradeoff parameter (=10^RADEXP).
- LATEXP (integer) - exponent of the latitudinal tradeoff parameter (=10^LATEXP).

#### Available Products

Dataseries for window functions and sections have primekeys T\_START and NDT. Except for the last two, all others have primekeys T\_START, LMIN, LMAX, and NDT. The pipeline also recognizes dataseries with the same structure and with the optional TAG primekey. The dataseries for inversions always have the primekey TAG. The last two dataseries have string primekeys as described below. JSOC series definition (jsd) files for these dataseries can be found at jsoc.stanford.edu/cvs/JSOC/proj/globalhs/data/ in the directory corresponding to the label that uses them (see below). Data that are not archived can generally be recreated by request.

- hmi.V\_sht\_1d (unarchived) - raw timeseries of length 1 day, chunked in l, usually retained online for 200 days.
- hmi.V\_sht\_72d (archived) - raw timeseries retiled to a length of 72 days with one l per record.
- hmi.V\_sht\_gaps\_72d (archived) - window functions of the above timeseries.
- hmi.V\_sht\_secs\_72d (keywords only) - sections of continuous data within each timeseries, used for subsequent detrending.
- hmi.V\_sht\_gf\_72d (archived) - detrended and gapfilled timeseries.
- hmi.V\_sht\_gf\_gaps\_72d (archived) - window functions of the above timeseries.
- hmi.V\_sht\_pow - power spectra of detrended and gapfilled timeseries.
- hmi.V\_sht\_modes (archived, permanent online) - ascii tables containing mode parameters fit using symmetric lorentzians.
- hmi.V\_sht\_modes\_archive (archived) - full results from all iterations of symmetric peakbagging.
- hmi.V\_sht\_2drls (archived, permanent online) - ascii tables of internal rotation, its errors, and resulting a-coefficients.
- hmi.V\_sht\_2drls\_asym (archived, permanent online) - ascii tables of internal rotation, its errors, and resulting a-coefficients.
  
- hmi.V\_sht\_gf\_retile (unarchived) - timeseries of various lengths retiled from hmi.V\_sht\_gf\_72d.
- hmi.V\_sht\_gf\_gaps\_retile (unarchived) - window functions of the above timeseries.

(COMING SOON! All of the above available for intensity as well.)

- hmi.vw\_V\_45s (archived, permanent online) - proxy of MDI medium-l data (vw\_V) made from HMI dopplergrams, which were binned by a factor of 4 and convolved with the same gaussian used for MDI. Used as input to make the following, in exact analogy the the dataseries above.
- hmi.vw\_V\_sht\_72d (archived)
- hmi.vw\_V\_sht\_gaps\_72d (archived)
- hmi.vw\_V\_sht\_secs\_72d (keywords only)
- hmi.vw\_V\_sht\_gf\_72d (archived)
- hmi.vw\_V\_sht\_gf\_gaps\_72d (archived)
- hmi.vw\_V\_sht\_modes (archived, permanent online)
- hmi.vw\_V\_sht\_modes\_archive (archived)
- hmi.vw\_V\_sht\_2drls (archived, permanent online)
  
- hmi.leakage (archived, permanent online) - leakage matrices. Two string primekeys, the first of which should always be omitted. For full resolution data, the second primekey is "fdreference". For the vw\_V proxy, it is "vwreference". Another entry will be created for intensity.
- hmi.eigenfunctions (archived, permanent online) - oscillation eigenfunctions used for inversions. One string primekey, so far only "default".

#### Data Formats

##### Timeseries

Timeseries are stored as two dimensional FITS files. For a single spherical harmonic degree (LMIN=LMAX), the dimensions will be 2\*NDT by LMIN+1. The datatype is float, but the data are actually complex, with the real and imaginary parts alternating in the file. For LMIN != LMAX, the second dimension will be (LMAX+1)(LMAX+2)/2 - LMIN(LMIN+1)/2.

##### Gap and Section Files

Gap files are a series of ones and zeroes stored as one-dimensional FITS files. The datatype is char and the length is NDT. A zero represents a data point that should be discarded. Section data are stored as keywords, but modules may instead take a text file as input. The first line of a section file is the number of continuous sections of data in the corresponding timeseries. Subsequent lines give the first and last timepoint, numbered from zero, of each section. A section file can be generated from a data record REC by "show\_info -q REC key=NSECS > file.txt; show\_info -q REC key=SECS >> file.txt".

##### Mode Parameters

Mode parameters are stored as ASCII tables. The fields are
degree, order, frequency, amplitude, width, background, x, {tan(asym)}, σ(frequency), σ(amplitude), σ(width), σ(background), σ(x), {σ(tan(asym))}, a1, a2, ..., aN, σ(a1), σ(a2), ..., σ(aN).
The parameter x is not fit for and is retained for historical purposes. The parameter tan(asym) and its error will not be present for fits with symmetric profiles. The value of N is either 6, 18, or 36. Any parameter with zero error has not been fit for (such as x).

##### Inversions

The segments of each record for 2d RLS rotational inversions include these files:
rot.2d, err.2d, splittings.out, and rmesh.orig.
The file rot.2d is an ASCII
table giving the rotation rate in nHz, and err.2d likewise gives the
error estimates for these values. The columns correspond to latitudes of
90-i\*15/8 where i is the column number, starting with zero. In other
words, every 8 columns correspond to 15 degrees, with latitude decreasing
to the right. The rows correspond to the radial mesh points, which are
given by taking every fourth value in rmesh.orig, which are given in fractional radius.

The file splittings.out contains an ASCII table that provides the a-coefficients
calculated from the inverted rotation rate.
The fields of this file are
degree, order, frequency, (ia+1)/2, 3, N/2, a\_ia\_in, σ(a\_ia\_in), a\_ia\_out, where ia is the a-coefficient index; only odd ia are used in rotational inversions.
As with mode parameters, the number of a-coefficients N is either 6, 18, or 36.

#### Modules

The global pipeline consists of six modules and one stand alone executable. Their source code can be found at jsoc.stanford.edu/cvs/JSOC/proj/globalhs/apps/ and the corresponding "libraries" can be found at jsoc.stanford.edu/cvs/JSOC/proj/globalhs/libs/.

- jv2ts  - takes images (usually dopplergrams) as input and outputs timeseries chunked in l. It provides the combined functionality of three historic modules: v2helio, helio2mlat, and qdotprod. It can optionally provide the output of the first two, namely longitude-sin(latitude) maps and these fourier-transformed in longitude and transposed.
- jretile  - input and output are timeseries, changes tiling in time and chunking in l.
- jtsfiddle  - detrends and gapfills timeseries. Output can simultaneously be timeseries, fourier transforms, power spectra, etc.
- jtsslice  - performs fourier transforms on a section of a timeseries to save on I/O. Output can be these and/or power spectra.
- jpkbgn  - extracts mode parameters from fourier transforms. Currently input are timeseries, output are ascii tables.
- inv2d.x - stand alone program to perform two dimensional RLS inversions for internal rotation.
- jrebinsmooth  - performs binning, gaussian smoothing, and subsampling of images.

#### Scripts

The pipeline is almost always run by the following scripts. They can be found at jsoc.stanford.edu/cvs/JSOC/proj/globalhs/scripts/.

- doglobalhs  - sets up working directory, environment variables, and runs and checks all subsequent scripts. This is typically the only script that is used at the commandline.
- dosht  - performs spherical harmonic transforms. Sets up and submits cluster scripts to execute jv2ts.
- doshtcheck  - checks that all the output expected from dosht is present (uses IDL).
- doretilen  - retiles input to multiple output time intervals. Sets up and submits cluster scripts to execute jretile. Jobs are divided in time, each one retiles all l.
- dodscopyn  (optional) - used to copy output of doretilen to another dataseries using dscp. Only needed for certain combinations of write slices and compression tiles, usually not used.
- doretile1  - retile input to a single output time interval. Sets up and submits cluster scripts to execute jretile. Jobs are divided in l, each one retiles the same interval in time. There is some overlap of functionality with doretilen.
- dodscopy1  (optional) - used to copy output of doretile1 to another dataseries using dscp. Only needed for certain combinations of write slices and compression tiles, usually not used.
- domkgaps  - examines timeseries for l=0,1,2,5,10,20, and 50 to construct window functions for raw timeseries (uses IDL).
- dogapfill  - performs detrending and gapfilling. Sets up and submits cluster scripts to execute jtsfiddle.
- dogfgaps  - examines one low-l timeseries to construct window functions for gapfilled timeseries (uses IDL).
- dopow  (optional) - performs fourier transforms and/or constructs power spectra. Sets up and submits cluster scripts to execute jtsfiddle. Only needed when output cover a different interval in time than the input.
- dopowslice  - performs fourier transforms and/or constructs power spectra for consecutive slices of the input timeseries. Sets up and submits cluster scripts to execute jtsslice.
- dopkbgn  - sets up and runs all iterations of the peakbagging, utilizing the scripts found in jsoc.stanford.edu/cvs/JSOC/proj/globalhs/scripts/pkbgn/. It calls the script doiter to set up and submit cluster scripts to execute jpkbgn.
- doinvert  - performs 2d RLS inversions. Does not use cluster, but rather executes inv2d.x on the local host.

The following scripts are not called by doglobalhs.

- dorebinsmooth  - sets up and submits cluster scripts to execute jrebinsmooth. Used to create vw\_V proxy from HMI dopplergrams.
- dorepeatpow  - used to regenerate fourier transforms and/or power spectra originally created using dogapfill and that have aged off disk. dogapfill itself should not be repeated since the gapfilled timeseries are archived. dopowslice, however, may be repeated.

#### Labels

The execution of the pipeline is controlled by parameter file templates. Each set of parameter file templates is assigned a label that refers to the set of all input parameters needed by all the modules and scripts for one particular way of executing the pipeline. Perhaps most significantly, the parameter file templates determine which dataseries are used as input and output for each of the modules. Therefore, each label describes a set of related data products, although some data products are shared between labels. Parameter file templates are located at jsoc.stanford.edu/cvs/JSOC/proj/globalhs/scripts/parmtemplates/.

- hmiv72d - default processing of dopplergrams, using symmetric lorentzians for the peakbagging. Peakbagging also takes into account horizontal displacement and the solar surface and distortion of eigenfunctions by differential rotation (the Woodard effect). Creates all data products ending in "72d" above and writes mode parameters to hmi.V\_sht\_modes.
- hmivret - retiles detrended and gapfilled 72 day timeseries generated by hmiv72d to timeseries of any length, creates power spectra from them, and runs the same peakbagging. This label has been used to analyze the time period covered by the last MDI Dynamics run in 2010 and may be used to analyze GONG time intervals. Writes to hmi.V\_sht\_gf\_retile, hmi.V\_pow, and hmi.V\_sht\_modes.
- hmiv72dasym - peakbagging performed using asymmetric mode profiles.
- hmivretasym - peakbagging performed using asymmetric mode profiles.
- hmivw72d - default processing of vw\_V proxy, using symmetric lorentzians for the peakbagging. Creates all data products beginning with "hmi.vw\_V" above.
- hmi\_vwv - used only for dorebinsmooth to generate hmi.vw\_V\_45s.
- hmiic72d - (COMING SOON!) default processing of continuum intensity images.

#### VERSION keyword

The VERSION keyword is typically propagated by the modules and scripts from the input to the output. However, at any point in the processing one may overwrite the VERSION keyword by amending the appropriate parameter file template. The data written by the label hmiv72d originally had VERSION="version0". However, when it was subsequently discovered that the error in the Carrington inclination was not taken into account in the spherical harmonic decomposition, they were reprocessed with VERSION="version1". As of November 2014, this is the current value for dataseries up to the mode parameters. These have been reprocessed again for a change in the application of the Woodard effect described above, and the new records have VERSION="version2". The old prescription used constant coefficients in the expansion of differential rotation, the new one calculated these coefficients from the fitted a-coefficients.

#### Examples

The official data products for the first 72 day time period could have been generated using the command
  
   
doglobalhs label=hmiv72d starttime=6328d totaltime=72d lmin=0 lmax=300 lchunk=80
  
   
but since the resulting data products are archived, this should not be repeated unless the processing changes. In that case, the corresponding parameter file templates should be amended to write a new value of the VERSION keyword. The parameters lmin and lmax could have been omitted, but here they are explicitly set to their default values for clarity. To create the high-l timeseries, we use
  
   
doglobalhs label=hmiv72d starttime=6328d totaltime=72d lmin=301 lmax=1000
  
   
where now lchunk is allowed to take its default value (40). For lmin>300, doglobalhs automatically skips the peakbagging. The parameter lchunk describes how many raw timeseries will go into one storage unit. See the documentation for doglobalhs for a full explanation of all its parameters.

  
  


---


**Downloaded 02 December 2017**
  
  


---
