# Supplementary material for: Global-Mode Analysis of Full-Disk Data from the Michelson Doppler Imager and the Helioseismic and Magnetic Imager
Source: Sol Phys. 2018 Jan 31;293(2):29. doi: 10.1007/s11207-017-1201-5 (PMC6566312; doi:10.1007/s11207-017-1201-5)
Supplement: Supplementary file 3 — (TAR 230.0 MB) [file 11207_2017_1201_MOESM3_ESM.tar › MDI/MDI_Global.html]

 


Joint Science Operations Center (JSOC) Data Products


Your browser does not support JavaScript, or has "active content" blocked; Menu system will not function.

Joint Science Operations Center (JSOC)
 


---

Match ALL words
Match ANY word

  
HMI Data Products

- HMI Observables
  - LOS Magnetograms
  - LOS Dopplergrams
  - Continuum Intensity
  - Linewidth
  - Linedepth
  - HMI Products Overview
- Magnetic Field Products
  - Magnetic Master Page
  - LOS Magnetograms
  - Vector Magnetic Field Data
  - Synoptic Maps
  - HARPS
- Helioseismology Products
  - Rings
  - Time Distance Products
  - Global Helioseismology Products
  - Farside Helioseismic Monitor
- Lower Level Data Products
  - Filtergrams

AIA Data Products

- AIA Data Products
- Level 1 Data- AIA Lev1 Overview

MDI Data Products

- MDI Observables
  - MDI Filtergrams
  - MDI Dopplergrams
  - MDI Continuum Intensity
  - MDI LOS Magnetograms
  - MDI Linedepth
- Magnetic Field Products
  - MDI LOS Magnetograms
- MDI Helioseismology Products
  - MDI Local Helioseismology Products
  - MDI Global Helioseismology Products
- MDI Calibration Data
  - MDI Calibration

SHA Data Products

- GONG Data
- TON Data

IRIS Data Products

- IRIS

SID Data Products

- SID

- Data Access
  - Getting Data
  - FAQ
  - Data products to Data series Map
  - Exportdata
  - Register Email
  - Lookdata
  - Script Driven
  - VSO
- Visual Catalog
  - Latest HMI Images
  - Latest AIA Images (Sun Today)
  - Browse HMI Images/Movies
  - Browse AIA JPEG2000 Images
  - Browse AIA Fits Images
  - HMI Image Gallery
  - AIA Image Gallery
  - MDI Image Gallery
- Docs
  - Wiki
  - AIA SDO Docs
  - AIA Data Analysis Guides
  - JSOC Overview
  - JSOC Software Doc
  - Software Repository
  - Problem Reports
- News & Events

## Global Helioseismology Products

*Global helioseismology is the study of resonant modes of
oscillation inside the Sun in order to infer properties of the interior.*

#### Algorithm for calculation

The global helioseismology pipeline begins with a spherical harmonic decomposition. MDI produced two types of dopplergrams that have been used as input: full disk dopplergrams (fd\_V) and vector-weighted dopplergrams (vw\_V). The full disk data take advantage of the full resolution of the MDI instrument: 1024x1024 with about 2 arcsecond per pixel. Due to telemetry constrains, however, these data are only available for a few months each year. The rest of the time the data have been convolved with a gaussian, subsampled by a factor of five in each direction, and severely cropped to yield the vector-weighted data with a resolution of about 200x200 pixels.

In either case, images are remapped to a uniform grid in longitude and sin(latitude), which provides an opportunity to make various geometric corrections. The resulting map is then apodized and an inner product is taken with a set of target spherical harmonic masks, yielding a complex amplitude as a function of spherical harmonic degree l and azimuthal order m for each image, up to l=lmax. For fd\_V, the apodization is performed from fractional image radius 0.90 to 0.95 and lmax=1000. For vw\_V, the apodization is from 0.83 to 0.87 and lmax=300. For each l and m a timeseries is constructed, typically of length 72 days, which are then detrended and gapfilled. Fourier transforms of these detrended and gapfilled timeseries are fitted to yield the mode parameters. The mode parameters can be inverted to yield the Sun's internal rotation and sound speed.

Since we cannot see the entire Sun, the spherical harmonic decomposition is not able to perfectly separate the modes. The extent to which each mode leaks into its neighbors is quantified by the leakage matrix. A separate leakage matrix is required for fd\_V and vw\_V, but in each case the same leakage matrix has been used for all times for the standard analysis.

For the full global helioseimology documentation (for developers), see here. For a complete scientific description of the pipeline, see this paper.

#### Keywords

Except for the last two dataseries below, all of the dataseries used in the globalhs pipeline have T\_START as their first primekey, which is typically slotted with a width of one day and an epoch of 1993.01.01\_TAI, the MDI epoch (see the DRMS dataset names documentation). When applicable, the next two primkeys will be LMIN and LMAX, although for some products in the pipeline these will be equal (they are retained for consistency among the dataseries). The last primekey will generally be NDT, the number of points in the corresponding timeseries, but dataseries may optionally contain a final primekey TAG, which is simply a string serving to label different processing options. For keywords not described here, see the JSOC keywords document.

- T\_START (time) - the beginning of the time interval a record corresponds to, specified as a date string or as an offset from the MDI epoch 1993.01.01\_TAI.
- LMIN (integer) - minimum spherical harmonic degree represented.
- LMAX (integer) - maximum spherical harmonic degree represented.
- NDT (integer) - number of time points represented.
- T\_STEP (float) - length of a time step in seconds, equal to the CADENCE keyword of the input data. This value is usually 60.0 and always a constant. The length of the timeseries is then NDT times T\_STEP.
- T\_STOP (time) - the beginning of the following timeseries, or T\_START + NDT \* T\_STEP.
- T\_OBS (time) - the midpoint of a timeseries as given by (T\_START + T\_STOP) / 2.
- MAPMMAX (integer) - maximum azimuthal order m in the remapping, usually constant.
- SINBDIVS (integer) - number of increments in sin(latitude) in the remapping, usually constant.
- MFLIPPED (integer) - a constant describing the sign convention for azimuthal order m: 0 for the MDI convention, 1 for the GONG convention.
- TAG (string) - an arbitrary string which serves to make records unique when their other primekeys are equal.
- VERSION (string) - an arbitrary string which facilitates searching obsolete versions of records.
- QUALITY (int) - a 16 digit bitfield specifying various aspects of data quality. Top bit set means no data.
- DATE (time) - the time at which the record was created.

Dataseries for inversions have these three primekeys as well:

- NACOEFF (integer) - number of a-coefficients used in fitting the mode paramters.
- RADEXP (integer) - exponent of the radial tradeoff parameter (=10^RADEXP).
- LATEXP (integer) - exponent of the latitudinal tradeoff parameter (=10^LATEXP).

#### Available Products

Dataseries for window functions and sections have primekeys T\_START and NDT. Except for the last two, all others have primekeys T\_START, LMIN, LMAX, and NDT. The pipeline also recognizes dataseries with the same structure and with the optional TAG primekey. The dataseries for inversions always have the primekey TAG. The last two dataseries have string primekeys as described below. JSOC series definition (jsd) files for these dataseries can be found at jsoc.stanford.edu/cvs/JSOC/proj/globalhs/data/ in the directory corresponding to the label that uses them (see below). Data that are not archived can generally be recreated by request.

- mdi.vw\_V\_sht\_72d (archived) - raw timeseries retiled to a length of 72 days with one l per record.
- mdi.vw\_V\_sht\_gaps\_72d (archived) - window functions of the above timeseries.
- mdi.vw\_V\_sht\_secs\_72d (keywords only) - sections of continuous data within each timeseries, used for subsequent detrending.
- mdi.vw\_V\_sht\_gf\_72d (archived, permanent online) - detrended and gapfilled timeseries.
- mdi.vw\_V\_sht\_gf\_gaps\_72d (archived, permanent online) - window functions of the above timeseries.
- mdi.vw\_V\_sht\_modes (archived, permanent online) - ascii tables containing mode parameters fit using symmetric lorentzians.
- mdi.vw\_V\_sht\_modes\_asym (archived, permanent online) - ascii tables containing mode parameters fit using asymmetric profiles.
- mdi.vw\_V\_sht\_modes\_archive (archived) - full results from all iterations of symmetric peakbagging corresponding to mdi.vw\_V\_sht\_modes.
- mdi.vw\_V\_sht\_modes\_asym\_archive (archived) - full results from all iterations of asymmetric peakbagging corresponding to mdi.vw\_V\_sht\_modes\_asym.
- mdi.vw\_V\_sht\_2drls (archived, permanent online) - ascii tables of internal rotation, its errors, and resulting a-coefficients.
- mdi.vw\_V\_sht\_2drls\_asym (archived, permanent online) - ascii tables of internal rotation, its errors, and resulting a-coefficients.
  
- mdi.vw\_V\_sht\_gf\_retile (unarchived) - timeseries of various lengths retiled from mdi.vw\_V\_sht\_gf\_72d
- mdi.vw\_V\_sht\_gf\_gaps\_retile (unarchived) - window functions of the above timeseries.
- mdi.vw\_V\_sht\_pow (unarchived) - power spectra of detrended and gapfilled timeseries.
  
- mdi.fd\_V\_sht (archived) - raw timeseries retiled one l per record (various lengths in time).
- mdi.fd\_V\_sht\_gaps (archived) - window functions of the above timeseries.
- mdi.fd\_V\_sht\_secs (keywords only) - sections of continuous data within each timeseries, used for subsequent detrending.
- mdi.fd\_V\_sht\_gf (archived, permanent online) - detrended and gapfilled timeseries.
- mdi.fd\_V\_sht\_gf\_gaps (archived, permanent online) - window functions of the above timeseries.
- mdi.fd\_V\_sht\_modes (archived, permanent online) - ascii tables containing mode parameters fit using symmetric lorentzians.
- mdi.fd\_V\_sht\_modes\_asym (archived, permanent online) - ascii tables containing mode parameters fit using asymmetric profiles.
- mdi.fd\_V\_sht\_modes\_archive (archived) - full results from all iterations of symmetric peakbagging.
- mdi.fd\_V\_sht\_modes\_asym\_archive (archived) - full results from all iterations of asymmetric peakbagging.
- mdi.fd\_V\_sht\_2drls (archived, permanent online) - ascii tables of internal rotation, its errors, and resulting a-coefficients.
- mdi.fd\_V\_sht\_2drls\_asym (archived, permanent online) - ascii tables of internal rotation, its errors, and resulting a-coefficients.
- mdi.fd\_V\_sht\_pow (unarchived) - power spectra of detrended and gapfilled timeseries.
  
- mdi.leakage (archived, permanent online) - leakage matrices. Two string primekeys, the first of which should always be omitted. For fd\_V, the second primekey is "fdreference". For vw\_V, it is "vwreference".
- mdi.eigenfunctions (archived, permanent online) - oscillation eigenfunctions used for inversions. One string primekey, so far only "default".

#### Data Formats

##### Timeseries

Timeseries are stored as two dimensional FITS files. For a single spherical harmonic degree (LMIN=LMAX), the dimensions will be 2\*NDT by LMIN+1. The datatype is float, but the data are actually complex, with the real and imaginary parts alternating in the file. For LMIN != LMAX, the second dimension will be (LMAX+1)(LMAX+2)/2 - LMIN(LMIN+1)/2.

##### Gap and Section Files

Gap files are a series of ones and zeroes stored as one-dimensional FITS files. The datatype is char and the length is NDT. A zero represents a data point that should be discarded. Section data are stored as keywords, but modules may instead take a text file as input. The first line of a section file is the number of continuous sections of data in the corresponding timeseries. Subsequent lines give the first and last timepoint, numbered from zero, of each section. A section file can be generated from a data record REC by "show\_info -q REC key=NSECS > file.txt; show\_info -q REC key=SECS >> file.txt".

##### Mode Parameters

Mode parameters are stored as ASCII tables. The fields are
degree, order, frequency, amplitude, width, background, x, {tan(asym)}, σ(frequency), σ(amplitude), σ(width), σ(background), σ(x), {σ(tan(asym))}, a1, a2, ..., aN, σ(a1), σ(a2), ..., σ(aN).
The parameter x is not fit for and is retained for historical purposes. The parameter tan(asym) and its error will not be present for fits with symmetric profiles. The value of N is either 6, 18, or 36. Any parameter with zero error has not been fit for (such as x).

##### Inversions

The segments of each record for 2d RLS rotational inversions include these files:
rot.2d, err.2d, splittings.out, and rmesh.orig.
The file rot.2d is an ASCII
table giving the rotation rate in nHz, and err.2d likewise gives the
error estimates for these values. The columns correspond to latitudes of
90-i\*15/8 where i is the column number, starting with zero. In other
words, every 8 columns correspond to 15 degrees, with latitude decreasing
to the right. The rows correspond to the radial mesh points, which are
given by taking every fourth value in rmesh.orig, which are given in fractional radius.

The file splittings.out contains an ASCII table that provides the a-coefficients
calculated from the inverted rotation rate.
The fields of this file are
degree, order, frequency, (ia+1)/2, 3, N/2, a\_ia\_in, σ(a\_ia\_in), a\_ia\_out, where ia is the a-coefficient index; only odd ia are used in rotational inversions.
As with mode parameters, the number of a-coefficients N is either 6, 18, or 36.

#### Modules

The global pipeline consists of five modules and one stand alone executable. Their source code can be found at jsoc.stanford.edu/cvs/JSOC/proj/globalhs/apps/ and the corresponding "libraries" can be found at jsoc.stanford.edu/cvs/JSOC/proj/globalhs/libs/.

- jv2ts  - takes images (usually dopplergrams) as input and outputs timeseries chunked in l. It provides the combined functionality of three historic modules: v2helio, helio2mlat, and qdotprod. It can optionally provide the output of the first two, namely longitude-sin(latitude) maps and these fourier-transformed in longitude and transposed.
- jretile  - input and output are timeseries, changes tiling in time and chunking in l.
- jtsfiddle  - detrends and gapfills timeseries. Output can simultaneously be timeseries, fourier transforms, power spectra, etc.
- jtsslice  - performs fourier transforms on a section of a timeseries to save on I/O. Output can be these and/or power spectra.
- jpkbgn  - extracts mode parameters from fourier transforms. Currently input are timeseries, output are ascii tables.
- inv2d.x - stand alone program to perform two dimensional RLS inversions for internal rotation.
- jrebinsmooth  - performs binning, gaussian smoothing, and subsampling of images.

#### Scripts

The pipeline is almost always run by the following scripts. They can be found at jsoc.stanford.edu/cvs/JSOC/proj/globalhs/scripts/.

- doglobalhs  - sets up working directory, environment variables, and runs and checks all subsequent scripts. This is typically the only script that is used at the commandline.
- dosht  - performs spherical harmonic transforms. Sets up and submits cluster scripts to execute jv2ts.
- doshtcheck  - checks that all the output expected from dosht is present (uses IDL).
- doretilen  - retiles input to multiple output time intervals. Sets up and submits cluster scripts to execute jretile. Jobs are divided in time, each one retiles all l.
- dodscopyn  (optional) - used to copy output of doretilen to another dataseries using dscp. Only needed for certain combinations of write slices and compression tiles, usually not used.
- doretile1  - retile input to a single output time interval. Sets up and submits cluster scripts to execute jretile. Jobs are divided in l, each one retiles the same interval in time. There is some overlap of functionality with doretilen.
- dodscopy1  (optional) - used to copy output of doretile1 to another dataseries using dscp. Only needed for certain combinations of write slices and compression tiles, usually not used.
- domkgaps  - examines timeseries for l=0,1,2,5,10,20, and 50 to construct window functions for raw timeseries (uses IDL).
- dogapfill  - performs detrending and gapfilling. Sets up and submits cluster scripts to execute jtsfiddle.
- dogfgaps  - examines one low-l timeseries to construct window functions for gapfilled timeseries (uses IDL).
- dopow  (optional) - performs fourier transforms and/or constructs power spectra. Sets up and submits cluster scripts to execute jtsfiddle. Only needed when output cover a different interval in time than the input.
- dopowslice  - performs fourier transforms and/or constructs power spectra for consecutive slices of the input timeseries. Sets up and submits cluster scripts to execute jtsslice.
- dopkbgn  - sets up and runs all iterations of the peakbagging, utilizing the scripts found in jsoc.stanford.edu/cvs/JSOC/proj/globalhs/scripts/pkbgn/. It calls the script doiter to set up and submit cluster scripts to execute jpkbgn.
- doinvert  - performs 2d RLS inversions. Does not use cluster, but rather executes inv2d.x on the local host.

The following script is not called by doglobalhs.

- dorebinsmooth  - sets up and submits cluster scripts to execute jrebinsmooth. Used to recreate vw\_V from full-disk dopplergrams.
- dorepeatpow  - used to regenerate fourier transforms and/or power spectra originally created using dogapfill and that have aged off disk. dogapfill itself should not be repeated since the gapfilled timeseries are archived. However, data that were created using dopow or dopowslice may be recreated by simply rerunning those scripts.

#### Labels

The execution of the pipeline is controlled by parameter file templates. Each set of parameter file templates is assigned a label that refers to the set of all input parameters needed by all the modules and scripts for one particular way of executing the pipeline. Perhaps most significantly, the parameter file templates determine which dataseries are used as input and output for each of the modules. Therefore, each label describes a set of related data products, although some data products are shared between labels. Parameter file templates are located at jsoc.stanford.edu/cvs/JSOC/proj/globalhs/scripts/parmtemplates/.

- mdivw72d - default vw\_V processing, incorporates all the considerations mentioned below, using symmetric lorentzians for the peakbagging. The Woodard effect is applied by computing the surface rotation from the fitted a-coefficients. Creates all data products ending in "72d" above and writes mode parameters to mdi.vw\_V\_sht\_modes.
- mdivwasym - runs peakbagging only, on the timeseries generated by mdivw72d, using asymmetric profiles. Writes mode parameters to mdi.vw\_V\_sht\_modes\_asym.
- mdivwret - concatenates detrended and gapfilled 72 day timeseries, creates power spectra from them, and runs peakbagging. This label has been used for the standard 360 day analysis, but is also used to analyze data contemporaneous with fd\_V timeseries or GONG time intervals. Writes to mdi.vw\_V\_sht\_gf\_retile, mdi.vw\_V\_pow, and mdi.vw\_V\_sht\_modes.
- mdivwasymret - typically used to run peakbagging only, on the timeseries generated by mdivwret, using asymmetric profiles. However, since its input is not archived, it may also perform the same initial retiling step as mdivwret. Writes mode parameters to mdi.vw\_V\_sht\_modes\_asym.
  
- mdifd - default fd\_V processing, using symmetric lorentzians for the peakbagging. Most data products, except power spectra, are archived, as described above.
- mdifdasym - runs peakbagging only, on the timeseries generated by mdifd, using asymmetric profiles. Results are archived.

The following labels have been used to test the application of various changes to the processing one by one. The default processing (mdivw72d) includes all these changes, with the exception that the last one is applied differently. These labels all use the same dataseries, which all have TAG as their final primekey, as described above. The TAG keyword is set to the label. JSOC series definition files for these dataseries can be found at jsoc.stanford.edu/cvs/JSOC/proj/globalhs/data/corr/. In order to repeat this type of processing, one must create the corresponding dataseries in a new namespace and amend the parameter file templates to use those series.

- corr1 - corrects for the change in plate scale over time.
- corr2 - corrects for cubic distortion in the MDI optics.
- corr3 - corrects for a P-angle error resulting from misalignment of the CCD.
- corr4 - corrects for an error in the Carrington inclination.
- corr5 - corrects for an alleged CCD tilt with respect to the optical axis of the instrument.
- corr6 - uses updated window functions and detrending.
- corr7 - uses an updated alogirthm for filling gaps.
- corr8 - includes horizontal component of the leakage matrix for the peakbagging.
- corr9 - adjusts leakage matrix to account for the distortion of eigenfunctions by the differential rotation (the Woodard effect). This presciption uses a constant expansion for the surface rotation.

The next set of labels have been used to investigate the effects of different apodizations and smoothing/subsampling. These analyses have only been run on the dynamics time intervals.

- mdifdvwap - processes full-disk data using the vw\_V apodization.
- mdivwfdap - uses the full-disk apodization on vw\_V data that were reconstructed from full-disk images.
- mdivwcomm - processes the vw\_V data with its regular apodization, but uses a window function common with the full-disk data as input to the gapfilling.

#### VERSION keyword

The VERSION keyword is typically propagated by the modules and scripts from the input to the output. However, at any point in the processing one may overwrite the VERSION keyword by amending the appropriate parameter file template. As of November 2014, the current records for the dataseries written by the label mdivw72d have VERSION="version0", up to the mode parameters. These have been reprocessed for the change in the application of the Woodard effect described above, and the new records have VERSION="version2". The reason the MDI data have no "version1" is because this string has been used for a reprocessing of the HMI data (see jsoc.stanford.edu/HMI/Global\_products.html). By skipping "version1" for MDI, "version2" can have the same meaning for both types of data.

#### Examples

The official data products for the first 72 day time period could have been generated using the command
  
   
doglobalhs label=mdivw72d starttime=1216d totaltime=72d lmin=0 lmax=300 lchunk=80
  
   
but since the resulting data products are archived, this should not be repeated unless the processing changes. In that case, the corresponding parameter file templates should be amended to write a new value of the VERSION keyword. The parameters lmin and lmax could have been omitted, but here they are explicitly set to their default values for clarity. The parameter lchunk describes how many raw timeseries will go into one storage unit. See the documentation for doglobalhs for a full explanation of all its parameters.
  
   
To (re)create a 360 day timeseries and power spectra, one could use
  
   
doglobalhs label=mdivwret starttime=1216d totaltime=360d laststep=pow lchunk2=20
  
   
The peakbagging should not be repeated since those results are archived.
  
   
To create the products corresponding to the label corr5 for the first 72 day period of 2004, one could run
  
   
doglobalhs label=corr5 starttime=4024d totaltime=72d
  
   
where lchunk has been left to take its default value (40).

  
  


---


**Downloaded 02 December 2017**
  
  


---
